# Supplementary material for: Gelsolin dysfunction causes photoreceptor loss in induced pluripotent cell and animal retinitis pigmentosa models
Source: Nat Commun. 2017 Aug 16;8:271. doi: 10.1038/s41467-017-00111-8 (PMC5559447; doi:10.1038/s41467-017-00111-8)
Supplement: Supplementary file 1 — Supplementary Information [file 41467_2017_111_MOESM1_ESM.pdf]

Title of file for HTML: Peer Review File
Description:

Title of file for HTML: Supplementary Information
Description: Supplementary Figures.

Title of file for HTML: Supplementary Data 1
Description: List of phosphoproteins (Column A) in Explorer array (Supp Fig 3) with phosphorylation ratios comparing diseased to control photoreceptors (HB02 v MB02 or H v M; Column B) and logged values (Column C).

Supplementary Information

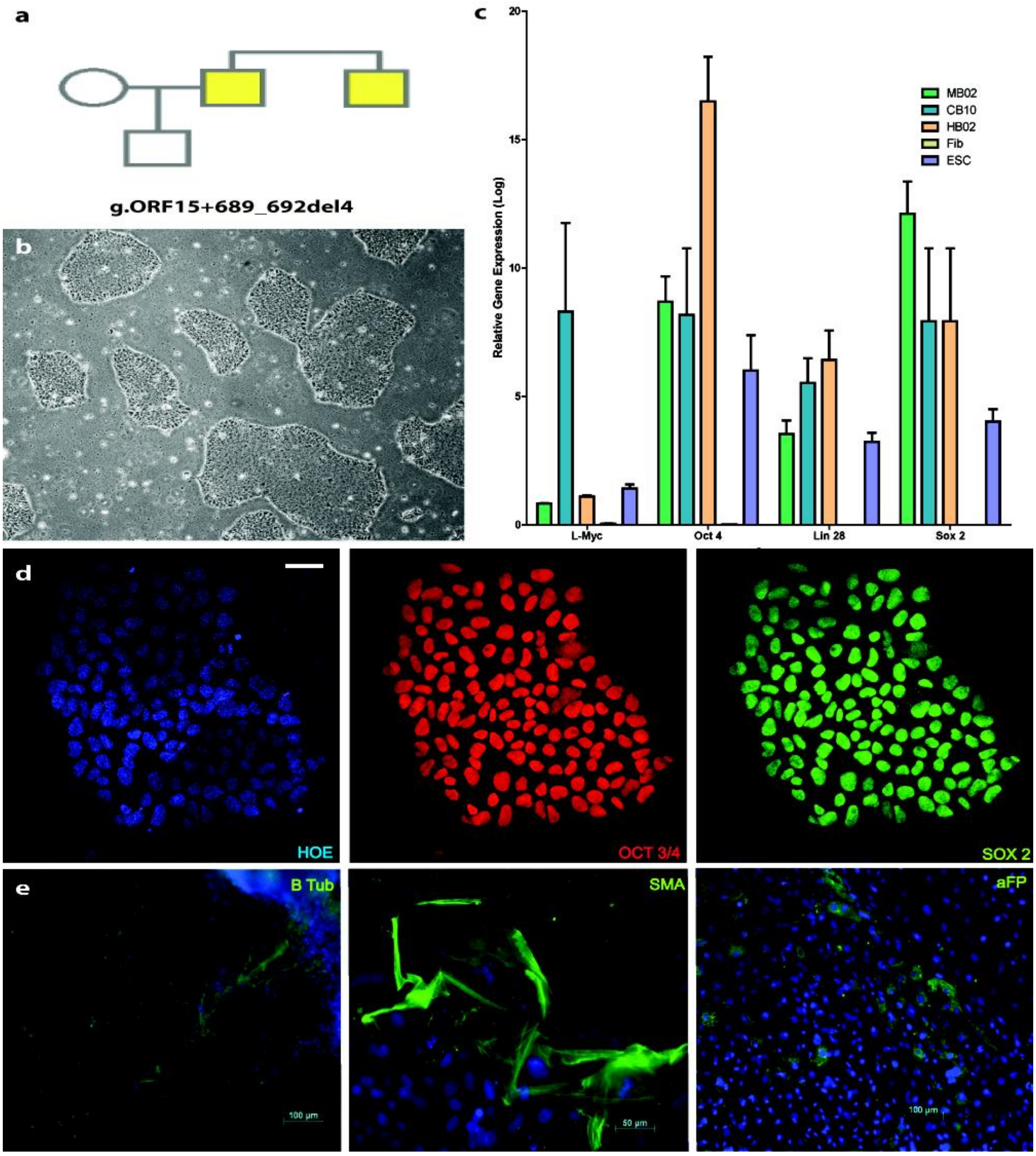

Supplementary Figure 1. iPSC generation from a family with an *RPGR* mutation. a: Two brothers with g.ORF15+689_692del4 mutations in *RPGR* and their unaffected son/nephew underwent skin biopsies. **b:** iPSC colonies were expanded following nucleofection with transgenes (see methods). **c:** qPCR demonstrated high endogenous expression of pluripotency markers in all cell lines used in this paper compared to fibroblasts (Fib) and the H9 embryonic stem cell line (ESC). **d:** Immunohistochemistry demonstrated the expression of pluripotency markers (OCT4, SOX2) in iPSC colonies. **e:** iPSC lines were pluripotent as demonstrated by their ability to differentiate down all three germ layers to form ectoderm (β-Tub staining), mesoderm (SMA staining) and endoderm (αFP staining). (Images representative of n=3 analysed). Scale bar: 10μm (d)

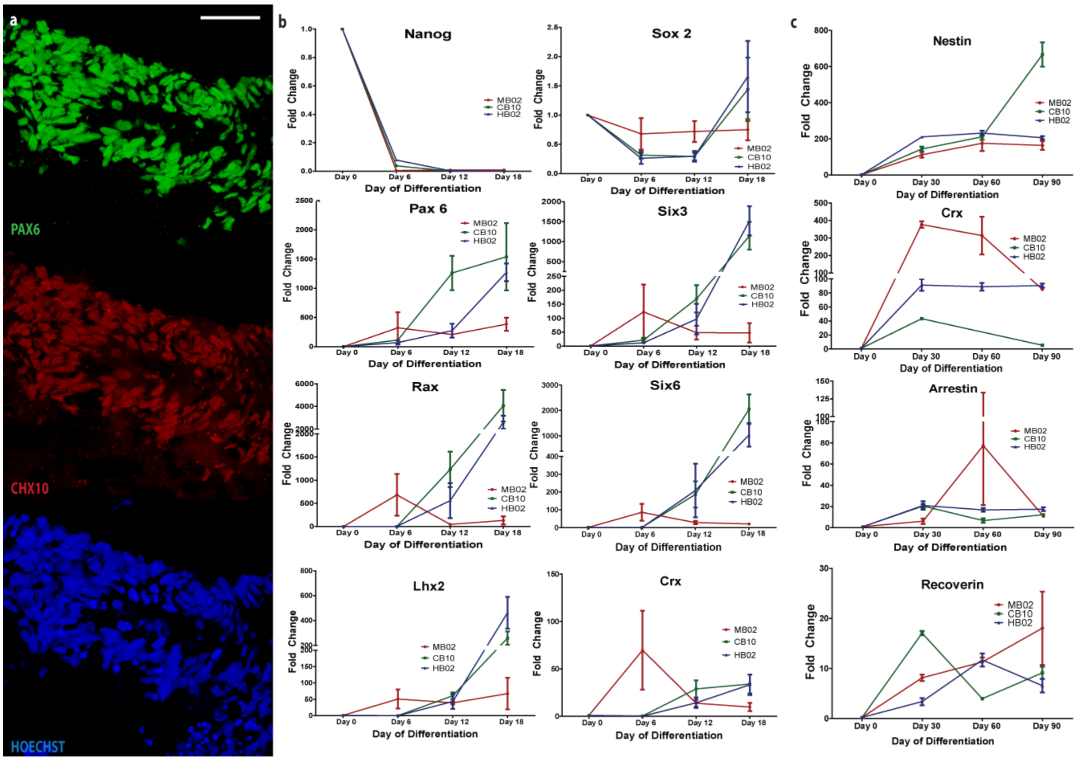

Supplementary Figure 2. Further characterization of iPSC derived 3 dimensional photoreceptor cultures. a: Successful patterning of free-floating aggregates resulted in PAX6^{+}/CHX10^{+} regions developing by day 10 (Images representative of n=3 analysed). **b:** qPCR demonstrated the loss of pluripotency in cultures, with *NANOG* downregulation observed by day 12 of differentiation. *SOX2* expression persisted and *PAX6* expression increased as cultures became neuralised. Upregulation of the eye field genes *RAX*, *SIX3*, *SIX6*, *LHX2* and *CRX* occurred in all three iPSC lines indicating successful eye field patterning. **c:** qPCR at later timepoints during photoreceptor differentiation showed persistence of neural markers (*Nestin*) alongside upregulation of mature photoreceptor genes (*Arrestin*, *Recoverin*). Scale bar: 20µm (a)

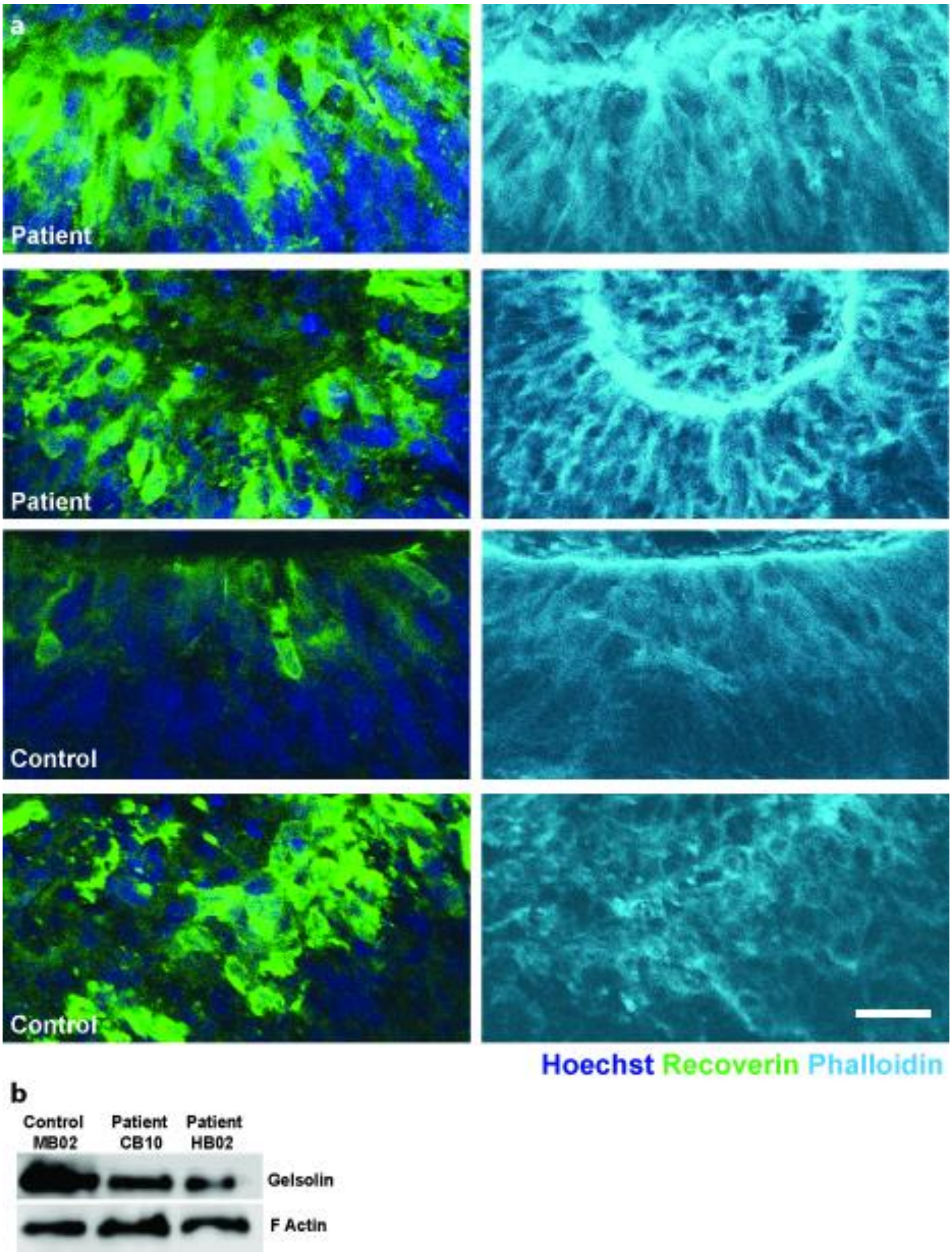

Supplementary Figure 3. *RPGR* mutant, iPSC derived, 3 dimensional photoreceptor cultures display perturbed actin regulation and reduced gelsolin activation. a: *RPGR*-mutant photoreceptors display increased actin polymerization, as evidenced by increased phalloidin staining in the recoverin-positive photoreceptors of patient-derived cultures (top 2 panels) as compared to photoreceptors from the control patient (bottom 2 panels) (Images representative of n=3 analysed).. **b:** F-actin-bound (active) gelsolin is reduced in *RPGR*-mutant photoreceptors, when normalized against actin.

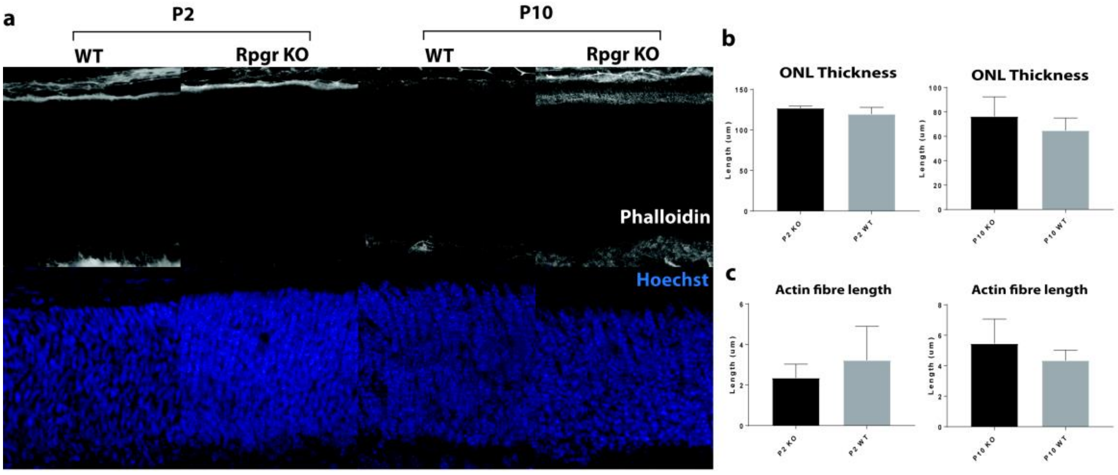

Supplementary Figure 4. The developing *Rpgr* KO mouse retina demonstrates no outer nuclear layer developmental abnormality or actin dysregulation prior to eyes opening. a,b: No outer nuclear layer (ONL - photoreceptor) abnormality is seen in the developing *Rpgr* KO mouse at post natal day 2 (P2) or 10 (P10) (compared to P2 and P10 wild type retina, respectively; Figures in **b** denote mean +/- SEM, one-way ANOVA). **a,c:** Actin bundles extending from the outer plexiform layer to the base of the connecting cilium are not significantly different in length at post natal day 2 (P2) or 10 (P10) in the KO mouse (compared to P2 and P10 wild type, respectively; Figures denote mean +/- SEM, one-way ANOVA). (Images representative of n=3 analysed).

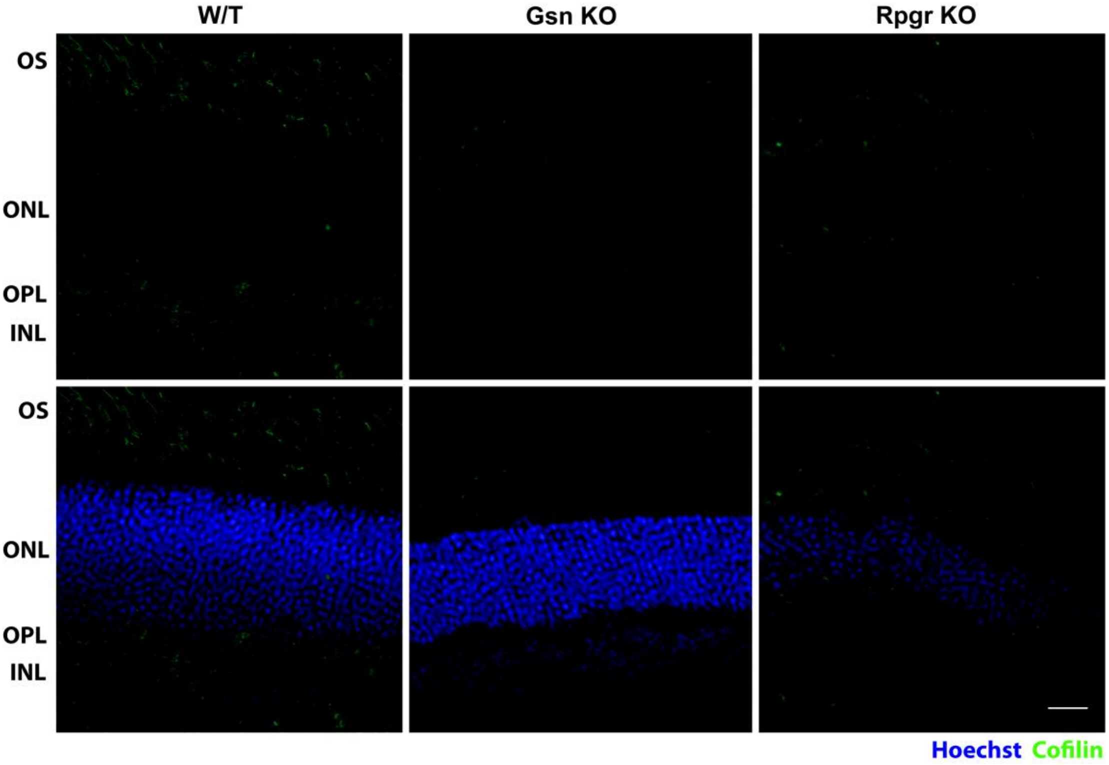

Supplementary Figure 5. Cofilin localization in the murine retina. Cofilin localizes to the photoreceptor outer segments (OS) and outer nuclear layer (ONL) in mature wild type murine retina. This localization is not altered in *Rpgr* KO or *Gelsolin* KO retina. (Images representative of n=3 analysed). Scale bar: 30μm

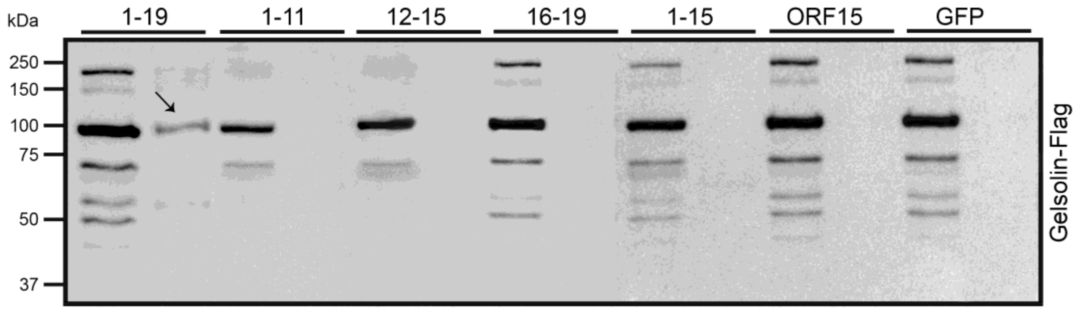

Supplementary Figure 6. Gelsolin directly interacts with the constitutive RPGR^{Ex1-19} splice variant. RPE1 cells transiently transfected with FLAG-Gelsolin and indicated GFP-RPGR encoding constructs were subjected to IP using GFP antibody. The precipitated proteins were analyzed by SDS-PAGE and immunoblotting using FLAG (antibodies. Cells expressing GFP tag alone were used as negative control. Arrow indicate specific immune-reactive bands.

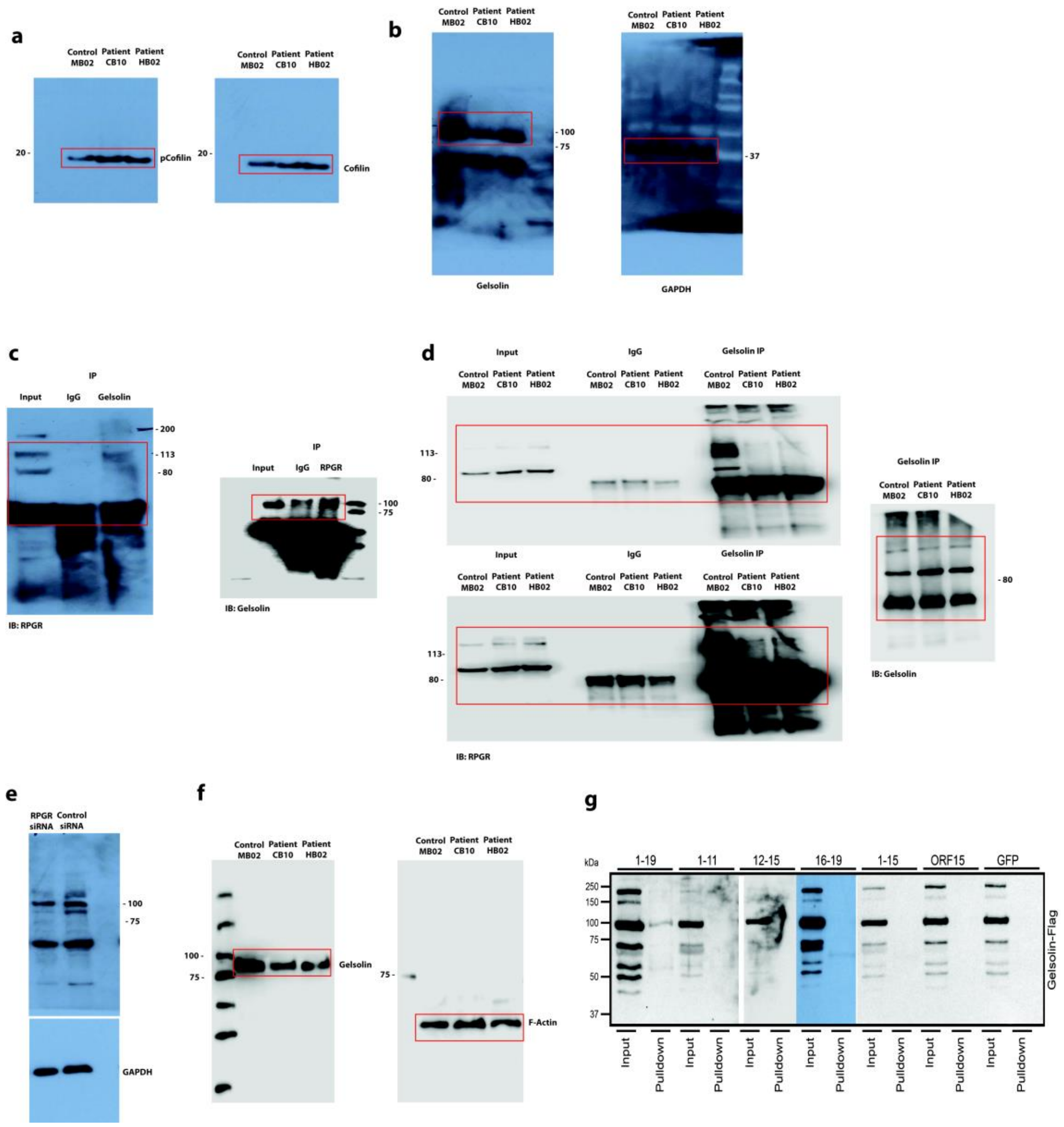

Supplementary Figure 7. Uncropped scans of the Western blot and Co-immunoprecipitation images seen in **a:** Figure 4b; **b:** Figure 4c; **c:** Figure 6a; **d:** Figure 6b; **e:** Figure 6c; **f:** Supplementary Figure 3b; **g:** Supplementary Figure 6.
